# ‘Sneaky’ Persuasion in Public Health Risk Communication

**DOI:** 10.1111/rati.12428

**Published:** 2024-11-04

**Authors:** Rebecca C. H. Brown

**Affiliations:** Uehiro Oxford Institute, University of Oxford, Oxford, UK

**Keywords:** manipulation, norms, persuasion, public health communication, relevance theory, risk communication

## Abstract

This paper identifies and critiques a tendency for public health risk communication to be ‘sneakily’ persuasive. First, I describe how trends in the social and health sciences have facilitated an approach to public health risk communication which focuses on achieving behaviour change directly, rather than informing people’s decisions about their health behaviour. I then consider existing discussions of the merits of informing versus persuading in public health communication, which largely endorse persuasive approaches. I suggest such accounts are unsatisfying insofar as their definitions of persuasion often fail to recognise its directional nature and the distorting effect this has on the total picture of the evidence. I re-characterise persuasion as directional influence aimed at achieving a particular outcome in the recipient and acknowledge that persuasive influence may also be manipulative. I then contrast this with (non-directional) information provision. I suggest that much persuasive public health risk communication is ‘sneaky’: it appears to be informative, but in fact presents a distorted picture of the evidence (in accordance with my characterisation of persuasion). I argue that such sneakily persuasive public health risk communication is unethical on the basis that it fails to adhere to the norms of cooperative communication.

## 1 Introduction: Agency In Health Behaviour And the Ecological Model of Health Promotion

Risk communication is a central activity of health promotion organisations. It has typically been thought of as benign, insofar as it largely involves information provision. Hence, public health communication has tended to receive relatively little attention from ethicists, in contrast to some other public health activities such as vaccination and screening programs, issues around social justice, or more abstract debates about the methods used in public health interventions (such as the role for coercion, nudging and other techniques).^1^ The Covid-19 pandemic sparked a little more interest in public health communication. Uncertainty and expert disagreement about the disease and effective mitigation measures made communication more challenging, while the necessity of implementing pandemic control measures made communication particularly important. Questions have been raised about the honesty of public health communicators during the pandemic, with suggestions that public health officials misled the public in their attempts to ensure people’s behaviour was more conducive to infection control (Prasad 2020; Powell and Prasad 2021; Intemann and de Melo-Martín 2023), or otherwise that they failed to update their beliefs based on the most plausible interpretation of available evidence and communicate this to the public (Lewis 2022).

In the context of non-communicable disease (such as cardio-vascular disease, cancer, diabetes, and stroke) public health communication has received attention for its role in the ‘responsibilisation’ of health—approaches to healthcare which emphasise individual responsibility and control regarding health and health behaviour (Brown 2018; Brown, Maslen, and Savulescu 2019). These discussions of risk communication have come as part of a more general analysis of the role of agency in health promotion. Trends in the social and health sciences have affected the way public health (and to some extent, public health communication) is seen, and a number of areas of work here have called into question the extent to which people can be seen as rational decision makers in the classical economic sense of being utility maximisers. In the 1970s, Amos Tversky and Daniel Kahneman initiated an influential area of research into the ‘heuristics and biases’ of individual psychology which described a number of ways in which people appear to err in their reasoning (i.e., fail to maximise expected utility or show consistent preferences) when faced with risky choices (Tversky and Kahneman 1974; Kahneman and Tversky 1979). Situationist psychology was developing around the same time and seemed to show that people’s behaviour was more a product of the circumstances they found themselves in than a result of robust character traits (Milgram 1963; Darley, Daniel, and Batson. 1973). These ideas found renewed popularity at the turn of the 21st century in the work of philosophers who posited that the empirical studies underlying situationist psychology represented a threat to existing philosophical accounts of agency and character (Harman 2000; Doris 2002).

These ideas soon filtered through into the policy space—including health—with the publication of *Nudge* (Thaler and Sunstein 2008) which described practical ways in which people’s tendency to be influenced by the local ‘choice architecture’ could be used to get people to make ‘better’ decisions. Alongside this was an enthusiasm for ‘social marketing’ strategies to promote health—the idea that the tools and techniques of marketing could be utilised to influence behaviour to improve health (Grier and Bryant 2005; Evans 2006). These public health strategies all ‘downplay’ the role of agency in shaping health behaviour. That is, they de-emphasise the importance of providing people with reasons to change their behaviour, or engaging them in deliberation, or encouraging them to reflect upon their behaviour, and focus instead on changing behaviour more directly. More support for these kind of agency-downplaying public health strategies came from research into the social determinants of health (Marmot et al. 2010). This work showed how health disparities were patterned across socioeconomic groups, with those most subject to social deprivation experiencing the worst health and those least subject to social deprivation experiencing the best health. One implication of this work has been taken to be that, since social deprivation plays such a central role in determining health outcomes, personal choice and responsibility are less important here than typically assumed (Wikler 2002; Venkatapuram 2013; Ahola-Launonen 2015; Bhugra 2017; Bognar and Hirose 2022).

The response to these various lines of research from the social and health sciences has been a tendency to criticise public health campaigns which rely upon encouraging individuals to make healthy choices (Ulijaszek and McLennan 2016; Friesen 2017; Brown 2018; Levy 2019). Instead, researchers have called for more focus on ‘system-level’ interventions (such as the regulation of food production and marketing, bans on alcohol and tobacco advertising, the use of defaults and taxation, etc.) to reduce unhealthy and promote healthy behaviours (Chater and Loewenstein 2022). We might call this tendency to focus less on the role of individual agency and instead emphasise the importance of social and environmental factors and group-level influences on health the ‘ecological model’ of public health promotion. It has received some level of buy-in from policy-makers (see examples such as the soft drinks industry levy introduced by the UK government in 2018) but probably less than advocates would like. The ecological model has been popular amongst those keen to counter the ‘individual responsibility’ narrative of health promotion and those who wish to place the onus on states and corporations to tackle chronic disease (Wikler 2002; Levy 2019; Tulatz 2019).

One effect of the ecological model of public health promotion has, I suggest, been a reduced emphasis on providing detailed and accurate information to people with the aim of informing their health behaviour. The provision of such information is only practically important if one assumes that individuals generally have the capacity to understand that information and translate it into behavioural changes. Yet the research described above provides reasons for thinking this might not be the case for many of the behaviours important for non-communicable disease, such as diet, physical activity, smoking and drinking. Many people do not change their behaviour in response to information that their current behaviour is likely to lead to health harms. At least some of the time, this might be because they cannot change their behaviour (e.g., a healthier diet might be prohibitively expensive; exercising regularly might be difficult if one is busy with work and caring commitments). In other cases, it could be because they have not understood the information (or its implications for their health and behaviour). Thus, barriers to behaviour change or understanding can render public health communication ineffective at promoting healthy behaviour. The ecological model of public health suggests that such barriers are particularly important and that, since individual agency has a limited role to play in health behaviour, it should have a limited role in health promotion.

In summary, there has been a relatively recent trend in public health analysis towards an ecological understanding of health behaviour and promotion. This model suggests that informational communicative strategies rest on a mistaken assumption that individuals have sufficient resources and capacity to change their behaviour in response to risk information. One implication of this model is that communicative strategies might best function as ‘nudges’ towards healthier behaviour. Rather than helping people to deliberate about their health and behaviour, so the thinking goes, public health communication will function better when it recognises—perhaps exploits—people’s biased, heuristic-driven thinking. On this model, the goal of public health communication is not (primarily or exclusively) to inform people’s decisions about their health and behaviour; it is to directly bring about behaviours that public health promoters think are worthwhile.

Whilst the ecological model of health promotion has clear merits (and is supported by some compelling empirical evidence), it has not, in fact, shown that people lack all control over their behaviour, or that they cannot comprehend risk information, or that they have no interest in making informed decisions about their health behaviour. I suggest the ecological model has been a little too successful in shifting emphasis away from individual responsibility for health, in the sense that it has facilitated a move towards risk communication that, rather than seriously attempting to inform people about their risks, provides risk information largely as a means of encouraging the adoption of public health recommended behaviours. It is not obvious that such communication is justified. To assess what ethical public health communication should look like, we need a better understanding of the legitimate goals of public health risk communication.

## 2 The Goals of Public Health Risk Communication

Public health communication often involves communicating to people about *risks*: how likely they are to suffer some health outcome; how their behaviour increases or decreases the likelihood of some outcome; what they can do to reduce their risk of harm, etc. It is this risk communication that will be the focus of my discussion.^2^

In the context of clinical medicine, the primary goal of risk communication is to support people to make autonomous decisions. Contemporary medical ethics emphasises the importance of respecting and supporting patient autonomy in decision-making by ensuring that patients’ decisions are informed and voluntary (GMC 2013; Neuberger, Hale, and Kerr 2015). Current best practice is described as ‘shared decision making’ whereby patients and their healthcare team collaborate to determine what course of action will best promote the values of importance to the patient. This is a departure from, on the one hand, ‘paternalistic’ models of decision making where clinicians essentially tell patients what treatment they will receive, whilst providing minimal information about alternatives, and on the other, ‘consumerist’ or ‘informed choice’ models where healthcare professionals’ role is restricted to providing the information with which patients are expected to make independent decisions (Cribb and Entwistle 2011). Attention to the details of how shared decision making should work has led to research to better understand, for instance, what formats of clinical information are most easily understood by patients, and discussions of how to identify and reflect patient values in clinical contexts (Cribb and Entwistle 2011; Entwistle, Cribb, and Watt 2012; Elwyn, Edwards, and Thompson 2016). Whilst there are undoubtedly barriers to shared decision making and limitations in the extent to which best practice is followed (Gravel, Légaré, and Graham 2006), there is at least buy-in from the healthcare profession that patients should be engaged (to the extent possible) with decisions about their care (General Medical Council 2020).

Public health communication, in contrast, is explicitly much more focused on outcomes rather than processes. This may partly be because of an important legal difference relating to informed consent. In clinical encounters, patients may be making decisions about whether or not to submit to an intervention which, absent informed consent, could count as an instance of assault or battery. As a result of clinical decision making, doctors *do things* to patients that patients must consent to have done to them. The decision making step is part of the informed consent process: it provides the opportunity for doctors to fulfil their obligations of disclosure and to ensure that patients understand (to a sufficient extent) to what they are consenting. Public health communicators have no parallel legal obligation for disclosure. When public health communicators recommend eating 5 portions of fruit and veg a day or exercising for 150 min per week they do not (physically) *do* anything to the recipient of this information, and there is no legal standard of disclosure that they must meet.

There is dispute about whether or not prevailing informed consent processes are apt for promoting patient autonomy (Manson and O’Neill 2007).^3^ But their prevalence reflects a recognition that patient autonomy is important and that information provision (of some form) is often essential for autonomy (Pugh 2020). Public health promotion is less focused on autonomy. The creation of the sub-discipline of public health ethics has even been seen as an opportunity to break free of the ‘dogma’ of autonomy that has dominated medical-ethical discussion (Dawson 2010). The goals of public health are generally understood to include promoting (average) population health, reducing health inequalities, equalising opportunities for health and promoting the health of the worst off groups (Munthe 2008; Brülde 2011; Holland 2022). Autonomy may be included here, either as another direct goal of public health activity, or as a constraint on public health interventions.

We might accept that public health promotion legitimately aims directly at promoting average population health or reducing health inequalities or some such, without also accepting that public health *risk communication* legitimately does these things. In fact, I think that public health risk communication should not be aiming directly at these goals, but should instead be aimed at informing people’s decisions about their health behaviour. In Section 4, I will suggest this is because communication aimed at informing people’s health behaviour is more consistent with the norms of cooperative communication than is (persuasive) communication aimed at achieving a particular outcome. Before presenting this argument, in the next section I will consider some existing discussions of the ethics of informing versus persuading in public health communication and refine the characterisations of persuasion and information provision that I will use.

## 3 Risk Communication: Informing Versus Persuading

In this section I will aim to clarify what should count as ‘persuasive’ versus ‘informative’ public health risk communication. I will summarise some of the existing ethical discussion of public health communication, where persuasive communication is commonly thought unproblematic. I will suggest this depends upon defining ‘persuasion’ in such a way that leaves out lots of instances of communication that I think we would typically call ‘persuasive’. I will offer a characterisation of persuasion and information provision that emphasises the intentions of the communicator and the ‘directional’ nature of persuasion as key to defining these forms of influence.

### 3.1 Manipulation Versus Coercion Versus Persuasion in Public Health Communication

There is some existing ethical discussion of whether or not public health communication may legitimately seek to persuade people about their health (and health-related behaviour). Authors here take their lead from philosophical accounts of persuasion which define it (roughly) as influence achieved through the provision of reasons (Faden and Beauchamp 1986; Blumenthal-Barby 2012; Tsai 2014; Mitchell and Douglas 2024). Persuasion is also often defined in contrast to manipulation and coercion, such that influence is *either* persuasive *or* manipulative *or* coercive (or something else). There is no accepted, unified account of manipulation, but it is variously taken to involve inducing the manipulated agent to make a mistake (Noggle 2020) or influence that involves trickery (Scanlon 1998); non-argumentative influence that bypasses or counters an agent’s reasons (Blumenthal-Barby 2012; Gorin 2014); influence that is non-rational or irrational (Raz 1986); or influence that exerts pressure on the agent (perhaps in the form of emotional blackmail or social shaming) which goes beyond persuasion but which stops short of coercion (Feinberg 1989).

Coercive influence, in turn, is often understood to be influence that applies extreme pressure on the agent (typically through the use of threats) (Wertheimer 1987). Whilst it need not cause the agent to make a mistake or act counter to her reasons or irrationally, it undermines the voluntariness of her actions.

Persuasion, then, is taken to be influence which neither manipulates (through inducing mistakes, using trickery, bypassing or countering reasons, etc.) nor coerces. Instead, persuasive influence operates through the provision of reasons which the agent can integrate into her deliberative reasoning processes. Persuasion is therefore assumed to respect one’s capacities as a rational agent by seeking to influence through these capacities, rather than bypassing or distorting them. Whilst manipulation and coercion are both typically thought to reduce or eliminate autonomy, persuasion is generally (though not exclusively) seen as autonomy-preserving or promoting (Fowler 1982; Arnold 2001; Greenspan 2003; Hausman and Welch 2010; Blumenthal-Barby 2012; Tsai 2014; Pugh 2020).

Given these assumptions about how persuasion differs from manipulation and coercion, it is unsurprising that ethicists have generally argued that persuasive public health risk communication is justified. Rossi and Yudell (2012), for instance, argue that persuasive communication is acceptable, emphasising that this holds so long as communication doesn’t coerce or control the agent (at which point it would presumably cease to qualify as persuasive influence anyway). Faden (1987) stipulates that for persuasion to be permissible it should not undermine the agent’s understanding. Oxman et al. (2022) also endorse persuasive communication, so long as its advantages outweigh its disadvantages, with the test being that well-informed people should agree with the justification for persuasion. A notable exception to this trend in support of persuasion is the (now closed) Winton Centre for Risk and Evidence Communication, whose motto was ‘To inform, not persuade’ (Winton Centre 2024).

In sum, then, persuasion is taken to be influence achieved through the provision of reasons that does not manipulate or coerce and hence is respectful of autonomy and an acceptable tool of public health communication.

### 3.2 What Does ‘Persuasive’ Public Health Communication Look Like?

One might accept that, on this picture, persuasive influence is indeed permissible, but nonetheless think these arguments are limited in their capacity to speak to real life examples of public health risk communication. Take the following fictional, though I think plausible, case:

#### Quit Smoking

Public health agency X wants people who currently smoke to quit. It publishes material that describes how smoking increases your risk of heart and lung disease. It asks smokers to imagine how devastated their loved ones will be if they die young; if their family has to watch them suffer through cancer treatment or struggle to breathe due to chronic obstructive pulmonary disease. It describes how much money it costs the health service each year to treat smoking-related conditions and lists a number of other things that could be funded with that money (better subsidised school meals, improvements to transport infrastructure, etc.). It details the decline of smoking over the last 50 years and how most people have now quit smoking. Accompanying the text are pictures of diseased lungs.

In this case, the public health agency provides the reader with reasons to quit smoking—it points to personal health harms, emotional distress to loved ones, financial costs to the health system, etc. But these reasons are presented in ways that might manipulate.^4^ For instance, the communication could induce mistakes (e.g., by causing people to believe that money saved on treating smoking-related disease will be spent subsidising school meals). By using emotional appeals about distress to loved ones, presenting disgusting imagery and leveraging social norms (that most people don’t smoke), the communication probably makes it more likely that recipients will view smoking (more) negatively, but it is not clear that recipients will have a more accurate understanding of the benefits and harms of smoking. These techniques could cause people to neglect the actual risks of smoking (and their magnitude), or to feel pressure to quit that is disconnected from the reasons they have to quit. Thus, such appeals plausibly induce irrational behaviour and/or act as reasons-countering or -bypassing (Raz 1986; Blumenthal-Barby 2012).

Such influence probably needs to be intentional to count as manipulation—is it? It is hard to establish, since this requires insight that we lack into the communicator’s mental states. But, as argued elsewhere (Brown and de Barra 2023), so predictable are some of these effects that it seems unlikely communicators would communicate in these ways if they wanted to be careful to avoid inducing mistaken beliefs, etc.

It could be countered that such techniques are likely to make people’s beliefs more accurate, not less, by making their reasons to quit more salient (e.g., by provoking emotional responses). I expect this is, at least sometimes, true. But it is hard to tailor such messages so that recipients will generally have appropriate emotional responses, which helps them to weigh their reasons correctly. *Quit smoking* seems to be an example of a communication campaign primarily designed to get people to quit smoking, regardless of whether or not it helps them form accurate beliefs about the benefits and harms of smoking.

Consider another example from McKenna (2024):

Craig is discussing treatment options for his chronic medical condition with his doctor. His doctor is a strong and persistent advocate of assistive technologies: he spends a lot of time trying to convince Craig of their virtues and little time on their downsides.

McKenna presents this as an instance of persuasion. Yet the reasons given to Craig are one-sided, such that the picture given by the total evidence is (intentionally) distorted by the doctor’s presentation. I suspect this is a common way in which persuasive communication operates: by being one-sided in the selection of reasons given.

In fact, empirical evidence shows that public health risk communication often selectively provides evidence and information is a way that is predictably likely to distort the overall picture of the evidence (including presenting evidence with more confidence than is warranted) (Sedrakyan and Shih 2007; Wegwarth and Gigerenzer 2011; Caverly et al. 2016; Brown and de Barra 2023; de Barra and Brown 2023). Public health communication about behaviours such as diet, physical activity, alcohol consumption, attending screening, and breastfeeding often provides reasons for adopting or avoiding particular health-related behaviours, but does so in ways that are likely to induce mistakes (e.g., create false beliefs), including mistakes about what reasons people have for adopting or avoiding particular behaviours. Put another way, it risks manipulating people through the provision of reasons.

One feature of persuasion that is rarely emphasised is that persuasion is directional (Coppock 2023).^5^ Persuaders are not neutral with regard to the beliefs formed / decisions made / actions taken by those they persuade.^6^ Persuaders provide reasons with the intention of getting the persuadee to form a particular belief / make a particular decision / take a particular action. As illustrated by the examples above, this will often involve selectively providing information so as to present more or stronger reasons for adopting the belief (etc.) that the persuader advocates, than for alternative beliefs. This means that persuasion will frequently risk manipulating, to a greater or lesser extent, insofar as it misleads people about the total evidence or reasons relevant in a particular context.

One response to noting the manipulative effects of such (directional) persuasion would be to re-classify much persuasive communication as manipulative. This would deviate significantly from ordinary usage (where we accept that being persuasive involves leaving out some information / emphasising other information, in order to slightly distort the overall picture and bring the persuadee round to our way of thinking). A better response, I suggest, is to accept that persuasion often at least risks being more or less manipulative, and instead to focus on describing another category of communication: information provision, which lacks the directional element of persuasion. We can then consider whether non-directional information provision is ethically preferable to (directional) persuasion.

I do not think it is helpful to insist that persuasive and manipulative influence are mutually exclusive. Communication which barely engages with reasons and relies heavily on mistake-inducing for its influence will be appropriately described as manipulative. Yet communication that presents (genuine) reasons, but does so in ways likely to induce mistakes—such as in the *Quit smoking* example and Craig’s doctor—should, I suggest, still qualify as persuasive whilst noting that it may also be manipulative. Whether or not an influence is predominantly persuasive or manipulative will depend on the degree to which it achieves influence via the engagement of reasons versus via inducing mistakes (or other manipulative effects such as trickery, reason bypassing or countering, etc.).

Given this, I will adopt the following characterisation of persuasive communication:

Persuasive communication brings about a change in attitude in the recipient via the directional giving of reasons.

Persuasion is directional because the persuader intends to produce a particular result (belief, decision, action, etc.) in the recipient and achieves this via the selective provision of reasons. The selective provision of reasons distorts the picture provided by the total evidence available.

The result aimed at by the persuader is not a full and accurate understanding of the matter at hand.

Persuasive communication is non-coercive: it does not utilise threats or exert overwhelming pressure on the recipient.

Persuasive communication can have features identified as manipulative: for instance it may foreseeably and / or intentionally induce a mistake, or exploit emotional vulnerabilities in the process of providing reasons.

Alongside this, I will characterise ‘information provision’ or ‘informative’ or ‘informational’ communication thus^7^:

Information provision involves presenting an accurate and balanced picture of the evidence within a domain as the communicator sees it.

The communicator is selective in which evidence she provides but this selection is guided by the communicator’s judgement as to what information the recipient would deem relevant, given his values.

Evidence is not selectively provided in order to achieve a particular attitude change in the recipient but in order to produce a full and accurate understanding of the matter at hand (or as close to this as is feasible).

The characterisation of information provision assumes a ‘curation’ model of risk communication (John 2020). Risk communicators must select what evidence and information to share, and they must ensure that this information is provided in a way that is comprehensible for recipients. Since they cannot provide *all* the information, they should selectively provide information that they deem relevant to the recipient based on their understanding of the recipient’s existing knowledge (gaps) and values (Bruine de Bruin and Bostrom 2013).^8^ Informative risk communication can include guidance or recommendations (since for many people, the judgement of public health professionals that, for instance, it is a good idea not to smoke or to consume only a small amount of alcohol, is likely to be relevant).

There is much more that could be said about how risk communicators can determine recipients’ values, how this should shape information provision, how information communication should be adapted to make it comprehensible to lay audiences, and so on. I shall not delve into these debates here since I would like to move on to consider why we should prefer information provision over persuasion as an aim of public health communication. One approach to making such a claim would be to argue that persuasive communication directly reduces autonomy or creates epistemic harms. I think this is very plausible and potentially an important implication of the account I offer here. However, this is not the approach I wish to currently pursue. Instead, I will suggest that risk communicators ought to avoid engaging in what I call ‘sneakily persuasive’ communication and should instead stick to informative communication in order to better adhere to communicative norms.

## 4 The Importance of Expectations

In this section, I will make the case that public health risk communication should not be ‘sneakily’ persuasive. Whilst public health promotion in general may permissibly aim at directly bringing about behaviour change, public health risk communication should be aimed at informing people’s autonomous decisions—decisions which reflect their values—as to their behaviour. This is, in part, because public health risk communication *appears* to be engaged in information provision and recipients reasonably expect it to be informative.

It is worth, first, saying a little about how communication operates and the importance of cooperative norms and shared assumptions to facilitate communication. By way of doing this, I’ll introduce an influential account of how communication works, relevance theory:

Human cognitive processes… are geared to achieving the greatest possible cognitive effect for the smallest possible processing effort. To achieve this, individuals must focus their attention on what seems to them to be the most relevant information available. To communicate is to claim an individual’s attention: hence to communicate is to imply that the information communicated is relevant. (Sperber and Wilson 1986)

Sperber and Wilson’s ‘relevance theory’ of how speakers and recipients send and receive information highlights just how essential cooperation is to communication. When we communicate we change the cognitive environment of the recipient in a way that provides either direct evidence of the information we wish to communicate, or, in the case of inferential communication, direct evidence of our intention to communicate some information. For instance, I might speak the words ‘I have a sore throat’ in a hoarse voice. The hoarseness of my voice directly communicates the soreness of my throat to the listener, whilst the meaning of the words I speak communicates my intention to tell her about my sore throat.

Much information is communicated not explicitly, but via implicature. This works because the communicator can make certain assumptions about how a change in the cognitive environment of the recipient will affect the recipient’s train of thoughts. For this to work, the communicator and recipient must have mutual knowledge of one another’s cognitive environment (i.e., the context within which the information is transmitted, received and communicative intent inferred). For instance, A might ask B ‘are you going for a picnic’ and B replies ‘it’s pouring with rain’. Whilst B’s reply might seem unrelated to A’s question, her response implicates that no, she is not going for a picnic. To understand this, one must understand that picnics are not much fun when it’s raining. Sperber and Wilson argue that implicature and inference are guided by an overarching principle of relevance: speakers will communicate in an optimally relevant way, such that an act of ostensive communication will communicate the most information with the least processing power required on the part of the recipient.^9^

With this in mind, let us consider the content and form of public health risk communication. Empirical research indicates that public health risk communication often uses tools to communicate that are likely to mislead. First, public health risk communication often leaves out relevant information, such as when it fails to acknowledge the expected magnitude of benefits people are likely to experience when engaging in health-promoting behaviours (Brown and de Barra 2023; de Barra and Brown 2023). The magnitude of such benefits is often quite small, and making it explicit risks reducing people’s motivation to engage in the (effortful, often somewhat unpleasant) behaviour.

Second, communication often emphasises the benefits of the behaviours that are recommended whilst downplaying their associated harms. This is sometimes done through ‘mismatched reporting’, whereby the benefits of an intervention are reported in relative risk format, whilst the harms are reported using absolute risks (Caverly et al. 2016). For instance, a drug might be described as reducing your risk of heart attack by 25% but carry a risk of liver injury of 0.1%. The first (big) number reflects a relative risk and the second (small number) an absolute risk. This tends to exaggerate the difference in probability of experiencing a benefit relative to a harm.

Third, communication sometimes exploits common errors of reasoning in ways likely to promote the formation of false beliefs. For instance, communication sometimes reports the total number of people who die from cancer in the context of recommending screening: this number, whilst typically large and frightening, does not tell the reader how likely screening is to reduce her chance of dying from cancer (which is often very small, if above zero) (de Barra and Brown 2023).

Finally, communication typically fails to acknowledge scientific uncertainty and gives the impression that the conclusions drawn from available clinical and epidemiological evidence are more confident than they in fact are (van der Bles et al. 2019; de Barra and Brown 2023). Such misleading practices of scientific communication also arise in the clinical evidence base, where they can hinder the correct interpretation of evidence by clinicians (Sedrakyan and Shih 2007; Wegwarth and Gigerenzer 2011).

What I want to suggest here is that these practices are particularly troubling insofar as they (a) result in persuasive communication while (b) appearing to function as information provision. That is, public health risk communication *looks* like it is providing an accurate and balanced picture of the evidence relevant to the recipient, given her values. Communicators are often at pains to emphasise the scientific credentials of the claims they make and to ensure the appearance of scientific rigour, neutrality, completeness, and trustworthiness.^10^ In fact, communicators often provide a selective picture of the evidence that appears designed to produce a directional influence on the recipient. We might call such persuasion which appears to look like information provision, ‘sneaky’ persuasion.

Communicating in this way flouts cooperative norms. Consider McKenna’s earlier example of Craig, whose doctor provides lots of reasons for adopting assistive technologies but fails to describe their downsides. This is a common form that persuasive influence takes—a one-sided (directional) provision of evidence in order to encourage someone to adopt a particular attitude. Relevance theory suggests Craig should assume his doctor is adhering to the principle of relevance when communicating the harms and benefits of assistive technologies. By failing to communicate any downsides of assistive technologies, the doctor implies that there are none (or at least, none of relevance to Craig). Even if the doctor thinks the downsides are not relevant (because, for instance, he thinks they are outweighed by the benefits) clinical consultations are guided by norms of disclosure which require doctors to allow patients to determine how to weigh up harms and benefits when making treatment decisions. Thus, all ‘material’ risks should be considered relevant.^11^

There is quite a lot of formal structure to make clear what the communicative norms are within clinical consultations, which set shared expectations amongst patients and clinicians regarding disclosure (what information is ‘relevant’ to communicate) (GMC 2013; Neuberger, Hale, and Kerr 2015). In the context of public health communication, however, there are no such formal requirements.^12^ This makes it harder to say just what the norms and expectations are when it comes to public health risk communication (and by implication, to say if and when communicators are flouting those norms). But I think the tone of the communication will often set expectations about the intentions of the communicator and thus shape the context within which the recipient interprets the communication. As discussed, public health risk communication often appears much like information one would expect in a clinical encounter: it appears quite balanced, it uses neutral, moderately scientific language (with an eye to being legible to those with low health literacy), it sometimes uses numbers to communicate benefits and harms, and it provides provisional recommendations about behaviour. This can be contrasted with public health communication which is clearly aimed at being persuasive: campaigns which stigmatise or shame behaviours such as smoking when pregnant or around children, drink driving, etc. These communications are explicitly persuasive (and may also be manipulative). Without suggesting there is nothing wrong with public health campaigns which seek to stigmatise and shame, they are, at least, transparent about their persuasive aims. Recipients of such communications can be under no illusion that the communicator is attempting to provide them with a balanced picture of the relevant evidence.

To recap: public health risk communication that uses sneaky persuasion appears informative (i.e., appears to provide a balanced and neutral picture of the evidence). In doing so, it sets expectations that it is not leaving out information that people are likely to find relevant when deciding how to behave, or presenting information in a manner that distorts the evidence and is likely to lead to false beliefs. It then fails to live up to these expectations: it does leave out relevant information; it does distort the evidence; it is intentionally directional in its influence.

I find it very plausible that some people do not expect public health risk communication to be balanced and non-directional. Surely, some expect public health communication to aim directly at behaviour change, rather than informing behaviour. Such people will presumably read all public health risk communication under the assumption it is persuasive, rather than informative. In such cases, the expectations of the recipient well be better aligned with the actions of the communicators, to the extent that they really do engage in persuasion. This seems parallel to other interactions where there is a general expectation of persuasion: for example, a used-car salesman is expected to highlight the car’s qualities to a customer and, to some extent, avoid mentioning its flaws. In contrast, someone selling a car to a good friend would be expected to be more candid.

Does the fact that some people expect public health promoters to act like used-car salesmen make sneakily persuasive risk communication permissible? I think it can mitigate the wrongfulness of this behaviour. To know how much it mitigates it would require empirical work to better understand the expectations of recipients of public health risk communication, and the intentions of communicators.^13^ But I think there is reason for being cautious about this defence. When there is ambiguity about people’s expectations, there is an obligation to err on the side of meeting the expectations of neutrality and balance rather than those of being directional and persuasive. In general, it seems worse to mislead when someone expects you to be honest than it is to be honest when someone expects you to mislead (think of the used-car salesman who is surprisingly candid versus the person who fails to reveal relevant information when selling his car to a friend). Given that at least some people will presumably expect public health risk communication to be informative rather than persuasive—particularly given the fact that sneaky persuasion encourages them to expect this—communicators should respect these expectations.

In this section, I have tried to show why sneakily persuasive public health risk communication is likely to flout the norms and expectations of cooperative communication. If correct, the above analysis suggests public health risk communicators defect when engaged in solving the collective action problem of communication: they appear to communicate informatively and exploit this appearance in order to change people’s beliefs and behaviour in ways conducive to their ends (promoting public health). Defecting on this cooperative enterprise is wrongful behaviour since it risks undermining the enterprise altogether: if sufficient people defect then communication becomes impossible. It is also untrustworthy behaviour and would be likely to lead to a loss of trust if uncovered.^14^ Much has been made of the need to maintain trust in public health institutions in order to secure public cooperation with, for instance, infection control measures. Yet this kind of behaviour in public health risk communication appears inconsistent with striving to be a trustworthy institution.

## 5 Concluding Remarks

Whilst the ecological model of health promotion has its merits, it has plausibly facilitated a shift away from informative public health risk communication. In this paper I have argued that public health risk communication should aim to be informative, rather than persuasive. It should provide an accurate, balanced picture of the evidence relevant to the recipient’s values. Many commentators on the ethics of persuasive public health communication conclude that persuasion is permissible, on the basis that it does not directly control the agent nor undermine her autonomy. I have argued, however, that the strength of these arguments is debatable and too dependent on defining persuasion in a way that excludes a lot of what we would typically call ‘persuasive’ public health communication, which will often involve being sneakily persuasive (insofar as the techniques of persuasive risk communication are subtly directional and maintain the appearance of information provision). Here, I have suggested that such sneakily persuasive communication violates communicative norms and that such a violation poses a broader threat to trust and cooperation.

I have not committed to a position on the strength with which we should criticise persuasive public health risk communication. Whilst I have argued that informative communication is preferable to persuasive communication, it is not obvious whether persuasive communication is generally impermissible, or what considerations would justify using persuasive communication. For instance, some will want to argue that in emergency contexts, it is permissible—and perhaps obligatory—to aim directly at behaviour change, and to do so in the most effective way possible. Public health is an arena where the stakes can be high. It seems plausible we could construct cases where the significance of ensuring particular behaviours amongst the public overwhelms the importance of respecting agents’ epistemic autonomy and preserving trust by respecting cooperative communicative norms. These are most likely to occur when large third party harms arise. Much public health risk communication, however, concerns the recipient’s own health. In such cases, it seems unlikely to me that persuasive communication—particularly of the sneaky variety—will be justified, and public health risk communicators should restrict themselves to being informative.

## Endnotes

1 Such a claim is hard to defend without substantial empirical engagement. But briefly: perusing the table of contents of a number of edited collections/monographs on public health ethics (Dawson 2011; Barrett et al. 2016; Mastroianni, Kahn, and Kass 2019; Holland 2022; Bayer et al. 2006; Anand, Peter, and Sen 2004; Beauchamp and Steinbeck 1999), one finds only one book with a chapter explicitly discussing health communication (Bernheim et al. 2013). A ‘model curriculum’ for ethics and public health, developed by a number of leading US-based bioethicists makes no explicit mention of health communication (Jennings et al. 2003). The Stanford Encyclopedia of Philosophy entry on Public Health Ethics lacks any mention of communication (Faden, Bernstein, and Shebaya 2022).

2 Public health communication also does other things. For instance, communicate to people about the availability of services. Some public health campaigns will be explicitly focused on, for instance, norm change (depicting a failure to wash hands after going to the toilet as disgusting; associating exercise with social fulfilment; etc.) I am focussing here on public health communication that is, in appearance at least, presenting risk information. Whilst a more comprehensive discussion should consider broader forms of public health communication, I think the clearest case against ‘sneaky’ persuasion can be made in relation to this apparently informative risk communication.

3 And indeed, whether autonomy ought to be given such a central role in clinical ethics (O’Neill 2002).

4 I say ‘might’ since one cannot guarantee the effect an influence will have on an agent. Particularly in discussing public health communication, we must be mindful that such communication reaches a broad audience, and will likely have different effects on different people.

5 An exception in the literature on the ethics of public health communication is Faden (1987).

6 It might be pointed out that those engaged in informing (where this is contrasted with persuading) are also non-neutral insofar as their intention is that the recipient form *true* beliefs or *informed* beliefs or some such. In this case, the difference between informing and persuading in relation to neutrality is one of degree—the non-neutrality of the persuader is more extensive than that of the informer, and the techniques she is willing to use extend to distorting the picture given by the total evidence.

7 There are parallels between what I describe as ‘information provision’ and what Manson and O’Neill call ‘good informing’. Manson and O’Neill criticise ‘mere’ information provision as including practices such as information dumping, and consider information provision insufficient to ensure recipients are actually *informed*.

8 I do not want to minimise how tricky this might be. The processes of judging people’s values, determining their existing beliefs, and identifying what information needs to be communicated (and how to do so) in order to achieve a full and accurate understanding will be both difficult and contested. Errors will inevitably be made. My contention is that communicators who are aiming to engage in ‘information provision’ (as I have characterised it) will make good faith attempts at this.

9 Another lens through which one might consider the ethical importance of communicative norms in public health is Grice’s (1975) maxims. Grice’s Cooperative Principle and maxims of Quantity, Quality, Relation and Manner also highlight the centrality of cooperation to communication, and offer norms for guiding that cooperation.

10 For instance, the health information website Healthline points to the fact that sources they cite are indexed by PubMed Central as indicative of their trustworthiness, yet this misrepresents the extent to which PubMed Central is an indicator of quality (it has, for instance, been shown to include articles from predatory journals) (Brown and de Barra 2023).

11 The *Montgomery* versus *Lanarkshire Health Board* case produced a judgement and new legal test for disclosure whereby doctors must provide patients with information about any material risks: risks to which a reasonable person in the patient’s position would attach significance (Neuberger, Hale, and Kerr 2015).

12 It will of course be embarrassing for the National Health Service if it is publicly shown to have failed to provide relevant public health information, or to have mislead people in its communications, but it will not be subject to legal charges of assault or battery, as could be the case if a doctor failed to provide accurate, relevant information to a patient prior to performing a medical intervention.

13 It is worth noting that this mitigation may be small. It results from the increase in cooperation that is possible when public health communicators act consistently, as people expect them to act (in this case, in persuasive rather than informative ways). If communications recipients have learned to expect public health communicators to always emphasise the harms of behaviours like smoking and never mention the benefits, then recipients will process communications accordingly. We might think of this in terms of ‘epistemic vigilance’ (Sperber et al. 2010): recipients can appropriately engage their mechanisms of epistemic vigilance, and protect their epistemic status, when communicators behave according to expectations. Recipients can, for instance, seek additional information about the benefits of health harming behaviours, if they are confident that communicators reliably disregard these effects. If communicators are aware of recipients’ expectations, and behave so as to conform to those expectations, then they knowingly support cooperation and are less subject to criticism for acting in a way that undermines cooperative communication.

14 This is rather speculative, but there is evidence that people are attuned to untrustworthy behaviour, and will withdraw trust in response (Mercier 2020).
